# Can APACHE II Score Predict Diabetic Ketoacidosis in Hyperglycemic Patients Presenting to Emergency Department?

**DOI:** 10.5812/aapm.21365

**Published:** 2014-09-01

**Authors:** Saeid Safari, Farzad Rahmani, Hassan Soleimanpour, Hanieh Ebrahimi Bakhtavar, Robab Mehdizadeh Esfanjani

**Affiliations:** 1 Department of Anesthesiology and Pain Medicine, Iran University of Medical Sciences, Tehran, Iran; 2 Emergency Medicine Department, Tabriz University of Medical Sciences, Tabriz, Iran; 3 Road Traffic Injury Research Center, Tabriz University of Medical Sciences, Tabriz, Iran; 4 Students’ Research Committee, Tabriz University of Medical Sciences, Tabriz, Iran; 5 Neurosciences Research Center, Tabriz University of Medical Sciences, Tabriz, Iran

**Keywords:** Diabetic Ketoacidosis, APACHE II, Emergency Department

## Abstract

**Background:**

Diabetic ketoacidosis (DKA) is an acute and life-threatening complication in diabetic patients. The current diagnostic criteria of DKA are metabolic acidosis, blood glucose level greater than 250 mg/dL and the presence of ketones in serum or urine. DKA patients referring to the emergency department (ED) are usually ill.

**Objectives:**

The present study aims to evaluate the efficacy of Acute Physiology and Chronic Health Evaluation II (APACHE II) scoring in predicting the critically illness in the hyperglycemic patients referring to the ED.

**Patients and Methods:**

We performed a prospective cohort study in an ED. One hundred eighty one patients older than 18 years with hyperglycemia were included in our study. Following the primary evaluation, the subjects were divided into DKA and non-DKA patients. APACHE II scores were calculated for all patients and then compared to each other. We determined predictive value, sensitivity, specificity and cut-off points of APACHE II score for DKA.

**Results:**

Sixty two patients had DKA. The comparison of APACHE II score among two groups of the patients did not show any significant difference (P = 0.597). There was no suitable cut-off point for APACHE II score to predict DKA.

**Conclusions:**

APACHE II score cannot be applied in the predicting of DKA in hyperglycemic patients admitted in ED.

## 1. Background

The patients with diabetes mellitus are faced with many acute complications including diabetic ketoacidosis (DKA) and hyperosmolar hypertonic non ketotic state (HHS) or chronic complications as vascular, renal etc. (1). DKA, defined by high levels of glucose, metabolic acidosis and ketonemia or ketonuria, is a life-threatening complication. These patients are usually critically ill and require intensive care (2, 3). APACHE II (Acute Physiology and Chronic Health Evaluation II) is a severity of disease classification system; one of several ICU scoring systems. It is applied within 24 hours of admission of a patient to an intensive care unit (ICU): an integer score from 0 to 71 is computed based on several measurements (4).

The APACHE II classification system is a version of a prototype system: APACHE. The basis for APACHE‘s development was the hypothesis that the severity of acute disease can be measured by quantifying the degree of abnormality of multiple physiologic variables (4). A study conducted by Freire et al. on APACHE II score among the patients in the ICU showed that the higher the score, the higher the mortality rate (5). A research done by Efstathiou et al. showed higher the APACHE II scores among the patients with DKA in the first day is associated with higher mortality rate (6). DKA leads into metabolic disorder in the body and this causes high score of APACHE among the patients. The existing studies applied APACHE II score to predict the mortality and hospital stay duration (4, 7, 8). Capnography was introduced as an alternative method to predict DKA in ED (9-11).

## 2. Objectives

The present study is aimed to use APACHE II score in predicting the critically illness in the hyperglycemic patients referring to the ED. Considering the fact that APACHE II score is routinely used in predicting the mortality as the outmost degree of critically illness in patients admitted in the ICU, we hypothesized that APACHE II score could also be used in predicting severity of illness in hyperglycemic patients (being involved with DKA) who are admitted for further evaluation in ED.

## 3. Patients and Methods

We performed a prospective cohort study of a convenient sample of patients in the ED of Imam Reza Medical Research and Training Hospital, Tabriz, East Azarbaijan, Iran. One hundred ten thousand admissions per year, during a four months period (December 2011 till March 2012) (12). Because of the lack a similar study, the present study was conducted as a pilot study on 181 patients. Patient collection was performed from 8 A.M. until 2 PM, seven days a week, while no sample collection was performed in the evening or night shifts. Inclusion criteria for the study were as follow: all adult patients older than 18 years old with hyperglycemia (BS ≥ 250 mg/dL) referred to the ED. Patients unwilling to participate in the study were excluded. This study was approved by the Ethics Committee of Tabriz University of Medical Sciences and registered under the Code Number 90104. On arrival, in all patients, serum glucose levels were measured by glucometer (Clever check, model TD 4209, SAN CHUNG, TAIPEI). Complete blood cell count, serum levels of sodium, potassium, urea and creatinine, urine ketone levels and arterial blood gases were measured. Patients with suspected DKA were further evaluated. DKA is characterized by serum glucose level > 250 mg/dL, ketonuria or ketonemia and metabolic acidosis (pH < 7.3 or Hco_3_ < 15 meq/L) (4). After the initial evaluations, APACHE II score was calculated for all the patients. Then, the scores of two groups (DKA and Non-DKA patients) were compared using SPSS (version: 17.0.1, SPSS Inc, Chicago, USA).

We used descriptive statistical approaches (domains, frequency, percentage, mean ± SD and variance). To compare the qualitative data, chi-square and to compare quantitative data, t-test and, if required, non-parametric Mann Whitney U tests were used. To define APACHE II score cut-off point in diagnosing DKA, Receiver Operating Characteristic (ROC) curve analysis) was used. In all cases, P value less than 0.05 was considered significant.

## 4. Results

**Figure 1.**
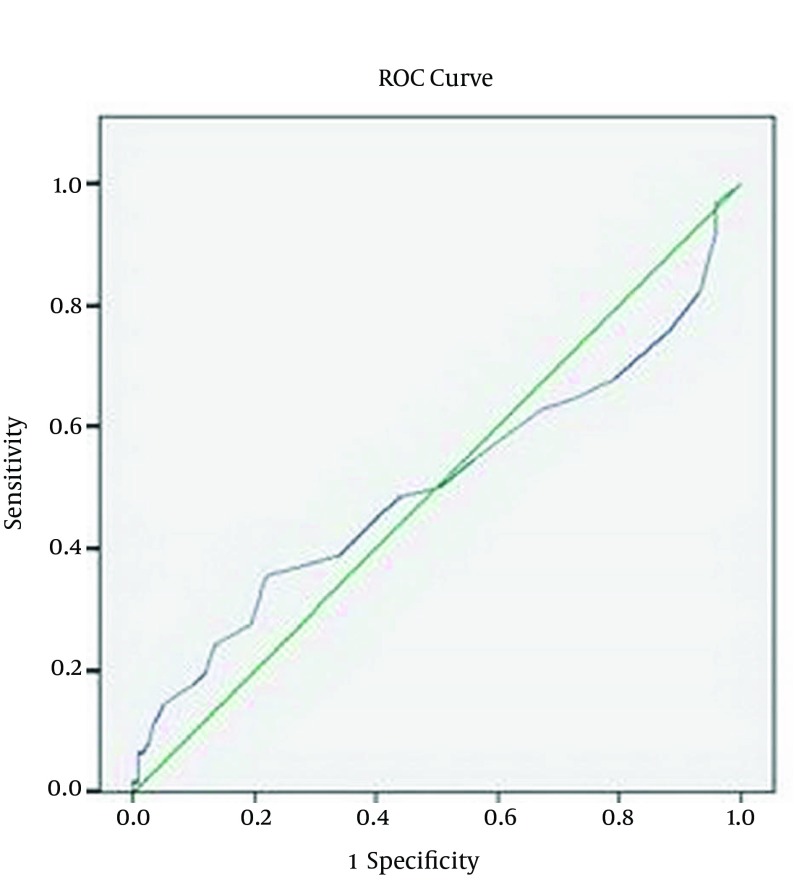
ROC Curve for Sensitivity and Specificity of APACHE II Score for Diagnosis of DKA

In the current study, one hundred eighty one patients including 107 females were studied. The mean age was 57.9 ± 17.8 years. Sixty two subjects had DKA while 119 patients had other conditions associated with metabolic acidosis. Table 1 shows that there was statistically significant differences between two groups (DKA and non-DKA) regarding age and serum glucose, but there was no significant difference in APACHE II score. Table 2 shows the comparison of the lab parameters including blood sodium, potassium, urea, creatinine, white cell blood, hematocrit and vital signs including the heart rate, respiratory rate, average arterial pressure and temperature in both groups of DKA and non DKA using non-parametric Mann Whitney U test. As is shown, the heart rate and average arterial pressure showed significant difference among two groups and the rest of the lab parameters did not show any significant difference. To obtain the sensitivity and specificity of APACHE II in predicting the critically illness in the hyperglycemic patients (DKA patients), ROC curves were conducted. The surface area under the curve is 0.503 (Figure 1). Because the low surface area and low sensitivity and specificity of the APACHE II score, a determination of the cut-off point was not possible.

**Table 1.** Demographic Characteristics, Laboratory Findings and APACHE II Score of Both Groups (DKA and non-DKA)

	DKA Patients	Non-DKA Patients	P Value
**Age, y**	51.01 ± 18.86	61.53 ± 16.13	0.001
**Gender**	23 males and 39 females	51 males and 68 females	0.454
**Serum glucoselevels, mg/dL**	458.66 ± 193.16	361.88 ± 92.94	0.001
**APACHE II**	13.01 ± 7.21	12.47 ± 5.10	0.597

**Table 2.** Laboratory Findings and Vital Signs of Both Groups (DKA and non-DKA)

Variables	DKA Patients	Non-DKA Patients	P Value
**Sodium (Na), meq/L**	137.08 ± 6.52	137.11 ± 5.94	0.670
**Potassium (K), meq/L**	4.68 ± 0.84	4.53 ± 0.48	0.710
**Urea, mg/dL**	35.80 ± 12.04	35.73 ± 10.65	0.965
**Creatinine, mg/dL**	1.18 ± 0.33	1.21 ± 0.35	0.510
**White blood cell,/mm** ^**3**^	11630.64 ± 5150.82	11021 ± 6103.74	0.172
**pH**	7.24 ± 0.13	0.07 ± 7.36	0.001
**Bicarbonate ion, meq/dL**	12.76 ± 4	3.61 ± 21.81	0.001
**PaCO**_**2**_	28.99 ± 7.92	6.74 ± 37.93	0.001
**Respiratory rate,/min**	21.16 ± 6.58	20.57 ± 4.80	0.885
**Body temperature,°C**	37.07 ± 0.72	37.18 ± 0.64	0.185
**Mean arterial pressure, mmHg**	86.93 ± 20.20	96.31 ± 17.11	0.002

## 5. Discussion

DKA leads into various metabolic disorders. The metabolic disorder affects APACHE II score and leads into obtaining high score among the patients (3). The current study aimed to apply this score to predict DKA among the patients with high serum glucose admitting ED. The results of the present study showed that there was no significant difference in APACHE II score among DKA and non-DKA patients. Various studies had been conducted on using APACHE II score to predict mortality rate, hospital stay duration or the need of the patient to be admitted in ICU. However, there is no study regarding the use of APACHE II score to predict DKA in hyperglycemic patients.

A study done by Freire et al. on 1506 patients in ICU showed that high APACHE II score in the first 24 hours after ICU admission predicted hospital mortality in an innercity MICU (5). A study done by Efstathiou et al. on the patients with DKA admitting ICU showed that the higher the score in the first 24 hours after ICU admission, the higher the mortality rate (6). A study conducted by Matic et al. on 129 patients regarding the selection of ventilator mode by APACHE II score showed that if the score > 20, the patients required aggressive method of mechanical ventilation; but if the score < 20, non-aggressive method should be applied (13). A study performed by Nfonoyim et al. on 30 patients with DKA showed that APACHE II score was suitable for decision making about the patient admission in ICU and it reduced the costs of wrong ICU admission (14). A study done by Chen et al. on 203 patients discharged from ICU showed that male gender and high initial APACHE II score in the first 24 hours after ICU admission were independent predicators of patient mortality after ICU discharge (15). A research done by Chiavone et al. on 521 patients in ICU showed that APACHE II score was useful for the severity classification of the disease but APACHE II score was a weak predictor of mortality rate of the patients (16). The only similar study to ours was the study of Lankisch et al. on the diagnostic value of APACHE II score in necrotizing pancreatitis. As no reliable cut-off point was determined in this study, the authors suggested that APACHE II score cannot be used in diagnosing necrotizing pancreatitis on the admission of the pancreatitis patients (17).

The present study applied APACHE II score to predict DKA among the patients with high serum glucose admitting to the ED. The results of the study showed that there was no significant difference in APACHE II variable among two groups of the patients (P = 0.597); because various variables including age, disease history, vital signs and lab parameters had crucial role in determining APACHE II score. Non-DKA patients had older age, the history of the diseases including hypertension, ischemic heart diseases, etc. and DKA group had low tachycardia, tachypnea, acidosis and blood pressure. The sum of the score of the variables was not different in two groups. The results of the study showed that there was significant difference in the heart rate and average arterial blood pressure in terms of the comparison of vital signs among two groups (P = 0.002); because the patients with DKA had unstable vital signs compared to non-DKA group. In the present study, there was no definite cut-off point for APACHE II score to predict the DKA among the patients with hypertension admitting ED.

The present study evaluated an alternative method to predict DKA and the results of the study showed that APACHE II score cannot be applied to predict DKA. We recommend to use the adjusted APACHE II score in the future studies by omitting age and disease history variables to predict DKA.
